# A snapshot of the chalkboard writing experiences of Bachelor of Education students with visual disabilities in South Africa

**DOI:** 10.4102/ajod.v8i0.523

**Published:** 2019-07-24

**Authors:** Roshanthni Subrayen, Rubby Dhunpath

**Affiliations:** 1 Disability Support Unit, School of Education, University of KwaZulu-Natal, Durban, South Africa; 2 Teaching and Learning Office, University of KwaZulu-Natal, Durban, South Africa

**Keywords:** Visual disabilities, teaching practice school placements, higher education, student retention, Bachelor of Education, teaching practice learning communities, stigma, chalkboard arrangements

## Abstract

**Background:**

South African higher education policy frameworks highlight renewed interest in equity, access and participation imperatives for students with disabilities (SWDs). However, students with visual disabilities continue to face barriers in their teaching practice school placements.

**Objectives:**

This article aims, firstly, to provide early insights into the barriers experienced by students with visual disabilities in their teaching practice school placements in under-resourced schools in KwaZulu-Natal, South Africa. Secondly, it introduces learning communities and a teaching practice pre-placement booklet to enhance equity, access and participation in teaching practice school placements.

**Method:**

This study adopted a qualitative methodology using semi-structured interviews to elicit data from two Bachelor of Education students with visual disabilities, who were part of a teaching practice learning community managed by the Disability Unit at the University. Thematic analysis was used, using Tinto’s Learning Community Model which generated valuable evidence to argue for institutional commitment to achieve equity, access and participation for students with visual disabilities.

**Results:**

Through engagement with a teaching practice learning community and a teaching practice pre-placement booklet, two students with visual disabilities responded to and managed the chalkboard in ways that promoted teaching and learning in the classroom. These retention support trajectories provide evidence to support enhanced equity, access and participation. Given the stigma associated with disability and the need for equity at policy level, higher education institutions should seriously consider systemic mechanisms for access, participation and success outcomes in the teaching practice school placements of students with visual disabilities.

**Conclusion:**

Barriers to participation signal the need for accessible teaching and learning strategies for use by students with visual disabilities in their teaching practice school placements. Teaching practice assessors should be alerted to contextual differences in resourced and under-resourced school settings and the diverse ways in which SWDs navigate these differences.

## Introduction

The United Nations Convention on the Rights of Persons with Disabilities (UNCRPD 2006) foregrounds the systemic discrimination experienced by people with disabilities and calls for equity, broadened access and participation^1^ in higher education (UNCRPD 2006). This injunction is mirrored in South African higher education policy frameworks (Republic of South Africa 1997a, 2001, 2018) with regard to broadening access and participation for students with disabilities (SWDs). Regrettably, these policy provisions do not resonate with the current experiences of individuals with disabilities. To explore this slippage, this article focuses on data derived from a qualitative research study with two Bachelor of Education students with visual disabilities, who were part of a teaching practice learning community managed by the Disability Unit at the University. The research focussed on their chalkboard experiences and how these influenced equity, access and participation as trainee teachers in their teaching practice school placements.

We begin by scanning the policy terrain on inclusion of SWDs in higher education in the United Kingdom (UK) and South Africa, followed by a review of the literature on the practice learning experiences of SWDs in these two countries. Thereafter, the context and background of this research study is documented. We move on to elucidate the theoretical orientation, which is informed by Tinto’s (2003, 2004) Learning Community Model for student retention and success. Following an overview of the research methodology, the results, discussion and conclusions are presented.

## The policy landscape: Rights of persons with disabilities

Foregrounding inclusion and the right to education for all, policy frameworks underscore equity and broadening access to enable the inclusion and participation of SWDs in higher education (Republic of South Africa 1997b, 2001, 2018; UNCRPD 2006). This is increasingly advanced as a basic human right and a social justice concern. The earnestness of these imperatives is reflected in equity, access and participation being included in institutional performance indicators to support social change and transformation. However, despite these policy advances, SWDs continue to experience complexities and tensions in negotiating fair and inclusive higher education. It has been suggested that these emerge because of control, hegemony and exclusion of persons with disabilities by social institutions (Terzi 2005).

The UNCRPD and the Constitution of the Republic of South Africa (1996) recognise disability as a human rights and social justice concern, denouncing all forms of oppression, exclusion and discrimination arising from disability. The UNCRPD’s (2006) assertion on higher education inclusion and participation for persons with disabilities provides for reform by stating in Article 24 (e) that ‘effective individualised support measures are provided in environments that maximize academic and social development, consistent with the goal of inclusion’. In South Africa, disability is considered as a system of discrimination requiring social redress and transformation through the imperatives of equity, access and participation in higher education (Republic of South Africa 1997b, 2001, 2018). Furthermore, the Integrated National Disability Strategy (1997c) and the Sustainable Development Goals (2016) draw our attention to the need for equity and inclusion in higher education for persons with disabilities. A more recent South African policy pronouncement, the Strategic Policy Framework on Disability for the Post-School Education and Training System (Republic of South Africa 2018), draws attention to the social inclusion of SWDs in higher education. This translates to the promise of universal design and curriculum accessibility; attention to violence, abuse and HIV and AIDS; inclusion of disability in higher education policies; access to sports, recreational and cultural activities; disability responsive retention and success outcomes and extensive disability sensitisation.

This study aligns to and acknowledges the distinctiveness of the social model of disability. This relates to the removal of social, attitudinal and universal design barriers and the importance of the individual and collective agencies of persons with disabilities to inform the design of inclusive social arrangements (Barnes 2007).

## Literature review

Studies have documented the challenges experienced by SWDs in higher education in accessing resources designed for able-bodied students (Gibson 2012; Opini 2012). These studies found that a complex and problematic relationship exists between SWDs and access to such resources. Our review of this literature found no international data on students with visual disabilities’ experiences of chalkboard access and participation. However, in South Africa, Subrayen’s (2018) study showed that the chalkboard produced complex and multi-dimensional power hierarchies, leading to the production of stigma.

Much rigorous research has been undertaken in the UK on the practice learning placements of SWDs. These studies highlight good practice models (Ashcroft et al. 2008; Botham & Nicholson 2014; Glazzard & Dale 2015; Griffiths 2012; Griffiths, Worth, Sculliard & Gilbert 2010), stereotyping and disempowerment (Glazzard & Dale 2015) and stigma and challenges arising from disability disclosure (Botham & Nicholson 2014). Interestingly, the country’s Equality Act (2010) provides guidelines for the preparation and design of practice learning placements for SWDs, and studies suggest that such models enhance retention, access and participation. Griffiths et al.’s (2010) case study of nursing SWDs argues for a comprehensive student-centric approach that supports collaboration with practice partners; disability disclosure; support systems; review of placement practices and institutional arrangements, all of which form the six-phase tripartite model. This model provides reflective learning, continuous assessment and evaluation suitable to individual student’s needs (Griffiths et al. 2010).

Botham and Nicholson’s (2014) modified action research study with physiotherapy SWDs corroborates Griffith et al.’s (2010) findings on practice partner engagement and on-going communication for open and early disability disclosure to support a structured practice placement procedure for evaluating and assessing clinical practice placements. This good practice model is also supported in a discussion paper by Ashcroft et al. (2008). These scholars assert that prior clinical training in simulated patient care laboratories for nursing SWDs improved clinical skills and enhanced readiness and confidence for clinical practice placements. Furthermore, Glazzard and Dale (2015) and Griffiths (2012) advocate for the use of specialised technology for trainee teachers with dyslexia. Their findings show improved retention and success rates in the trainee teachers’ teaching practice school placements. As noted by Glazzard and Dale (2015), tutors trained in dyslexia and support plans for teaching practice reduced barriers in such placements.

Glazzard and Dale (2015) and Botham and Nicholson (2014) agree that inequities and complexities exist in practice learning placements for SWDs in the UK. The results of Glazzard and Dale’s (2015) study show that trainee teachers with dyslexia experienced stereotyping, stigma and disempowerment by school mentors and tutors in their teaching practice school placements. This negatively influenced teaching and learning in the classroom context. Another important finding was that SWDs delayed disability disclosure in singular or multiple practice learning placements for fear of discrimination leading to stigma (Botham & Nicholson 2014; Stanley et al. 2011). Hence, disability disclosure is fraught with tensions and complexities where SWDs have to consistently negotiate and re-negotiate decision-making related to the timing of disability disclosure (Botham & Nicholson 2014; Stanley et al. 2011). However, despite this rigorous research on practice learning placements in the UK, these studies do not offer directions on strategies to enhance equity, access and participation for Bachelor of Education students with visual disabilities in their teaching practice school placements.

In comparison to the UK, little progress has been made in South Africa on the practice learning arrangements for SWDs in higher education. Ndlovu and Walton (2015) recently noted that there is a paucity of evidence to highlight the unique experiences of SWDs in their practice learning placements. This presents a challenge in critically understanding and analysing the practice learning experiences of SWDs in South African higher education. There is, thus, a need for a critical examination of the ways in which SWDs negotiate their teaching practice learning experiences in the school context. Small-scale data sets suggest that South African SWDs are experiencing tensions in these placements (Ndlovu & Walton 2015; Ntombela & Subrayen 2013; Subrayen 2011, 2018).

Van den Heever’s (2017) study at a higher education institution in South Africa highlights the clinical experiences of final year able-bodied nursing students. These students acquired psycho-social disabilities, such as continuous stress and anxiety, chronic physical and emotional fatigue and personal identity detachment, while in their clinical training with young traumatised children in hospitals. These acted as barriers to access and participation in these students’ clinical nursing practice placements (Van den Heever 2017). A qualitative study in KwaZulu-Natal found that academic staff’s lack of awareness of students with visual disabilities and unique chalkboard access trajectories led to the exclusion of a student with a visual disability from entering a Bachelor of Education programme (Subrayen 2011). This constrained retention and success of students with visual disabilities, hence violating their freedom and agency (Sen 1999) to do and be what they have ‘reason to value’ (p. 87).

Two other studies found that Bachelor of Education students with visual disabilities experienced constraints in their teaching practice school placements. Ntombela and Subrayen’s (2013) situational analysis concluded that blind students experienced barriers related to the lack of human support and specialised assistive technologies to navigate their teaching practice school placements. This impacted on their retention and success outcomes. The authors argued that these exclusions provide evidence to support the argument that students with visual disabilities experience barriers to chalkboard access. Institutional commitment is, thus, required to consider visual disability as human diversity in the apportionment of accessible resources for teaching practice school placements.

More recently, Subrayen’s (2018) sociological analysis of the experiences of Bachelor of Education students with visual and physical disabilities found that these students experienced many inequities in their teaching practice school placements. Complex and multiple power hierarchies lead to the production and reproduction of stigma, and a lack of freedom and agency to make decisions about teaching practice, environmental barriers in the teaching practice context and the chalkboard as a normative resource prevented the achievement of equity for eventual equality. As observed by Terzi (2005), normative resources are designed by social institutions without accounting for the social realities of persons with disabilities. These resources serve to exclude persons with disabilities (Terzi 2005) and constrain individuals’ human development and expansion of their capabilities (Sen 1999). These South African studies highlight that despite the invocation of equity, access and participation imperatives in higher education policy frameworks (Republic of South Africa 1997b, 2001), SWDs continue to experience systemic institutional constraints relating to stigma, inadequate student funding, marginalisation and a lack of human support and specialised technological software (Ndlovu & Walton 2015; Ntombela & Subrayen 2013; Subrayen 2018). However, Subrayen’s (2018) study also demonstrated that learning communities (Tinto 2003, 2004) for student engagement supported equity, access and participation in teaching practice placements.

This section has demonstrated that, in both the UK and South Africa, there is documented evidence on the constraints confronting SWDs in their practice learning placements. However, there remains a need for a deeper sociological understanding of the redress and transformative measures relating to the chalkboard experiences of students with visual disabilities.

## Context and background

The School of Education at the University was the site for the research. The core teacher education curriculum requires trainee teachers to participate in a compulsory 16-week practice teaching experience in a school setting. This practical exposure is intended as a catalyst for nuanced and diversified school-based experiences to enable trainee teachers to become reflective practitioners. The School of Education’s Disability Support Unit’s primary mission is to ensure academic integration and support for SWDs. A student group programme undertaken by the Unit involves group collaboration and sharing experiences of access and participation in teaching practice school placements.

Arising from these programmes, the lead author, in her capacity as the University’s Disability Coordinator at its School of Education, developed a booklet entitled ‘Management Strategies for Effective Teaching Practice Placement for Students with Visual Disabilities’. The booklet was formulated through the voices of SWDs on their teaching practice school placement experiences and is used as a critical pre-placement teaching practice tool. Prior to embarking on his or her teaching practice school placement, a copy of this booklet is provided to each SWD to guide their access, participation and retention in their placement. It is presented in the requisite print accessibility format such as large print in the required font size, Braille or through voice synthesised software.

The first part of the booklet details the challenges SWDs experience in their teaching practice school placement. These include personal barriers; the lack of reasonable accommodations at the placement schools; universal design barriers; attitudinal challenges emanating from learners and school staff; challenges arising from the disclosure or non-disclosure of disability and chalkboard inaccessibility. In the second part of the booklet, the ways in which SWDs navigate their teaching practice school placement to ensure retention and success are documented. Intervention strategies related to social skills training; classroom management; learner engagement; specialised technological software and reworking traditional methods of the chalkboard; consultations with teaching practice assessors and school mentors and the advantages of disability disclosure are also documented. Furthermore, the booklet provides advice from a professor who is blind on the requisite psychological processes for effective teaching and learning in the classroom.

Ideas such as collaborative and cooperative learning; sharing of knowledge; engagement with common themes; mutual connectedness and the co-construction of skills and competencies from Tinto’s (2003, 2004) Learning Community Model are used during the teaching practice school placement programmes. Within-group collaboration, support and sharing of experiences and students’ engagement with the booklet are underpinned by Tinto’s (2003, 2004) Learning Community Model for student perseverance, retention and success, as explicated below.

## Theoretical orientation

Tinto (2003, 2004) defines learning communities as a structure for beginning, undecided and academically developing students. We draw on Tinto’s theoretical framework to emphasise the potential of appropriate support for retention and success for SWDs, particularly in the context of promoting collaborative and cooperative learning through linked courses, sharing and discussion of curriculum content. Furthermore, he argues that learning communities are structured in ways that connect students with one another by organising common themes which gives meaning to their mutual connectedness. Importantly, Tinto (2003) posits that learning communities share three commonalities. The first is sharing knowledge in a focussed and coherent manner to establish deep levels of dialogue. The second commonality relates to shared knowing through the enrolment of the same students in the learning communities to facilitate their involvement and engagement, and co-construction of academic skills and knowledge to enhance deep levels of dialogue and the development of their cognitive abilities. The third commonality is shared responsibility, wherein there is a responsibility to every voice, hence enhancing collaboration and co-operation to improve and develop the entire learning community collectively. Although Tinto’s (2003, 2004) Learning Community Model does not provide explanations on shared knowledge, shared knowing and collaborative and cooperative strategies as they relate to visual disabilities in the context of teaching practice school placements, his work highlights sustained academic engagement and integration. Hence, Tinto’s (2003, 2004) model has the potential to promote critical engagement in broadening access and participation for students with visual disabilities. This theoretical lens is valuable as it stimulates arguments for institutional commitment. However, Tinto (2003) claims that the value of learning communities is based largely on anecdotal evidence, self-reports and assessments resulting in institutions working without any clear direction. The few available sociological studies that are positioned in Tinto’s model tend to focus on able-bodied students in relation to socio-economic status and curriculum concerns. As will be demonstrated later, we use requisite evidence to argue that learning communities do promote and enhance retention and success for students with visual disabilities.

## Research methodology

The enquiry aimed to explore two key research questions: (1) What are the chalkboard experiences of Bachelor of Education students with visual disabilities in their teaching practice school placements? (2) How do these students negotiate their chalkboard access to enable teaching and learning in the classroom? A qualitative approach provided tools to deepen our understanding and interpretation of the unique experiences of students with visual disabilities in their teaching practice school placements at the University. Data were obtained through qualitative research instruments, as the researchers were interested in capturing the participants’ authentic voices (Denzin & Lincoln 2005).

Non-probability sampling was used because the lead researcher is the Disability Coordinator at the said university and is the only staff member responsible for the support and management of all SWDs. This sampling strategy allowed for what Bryman (2012) considers ease of ‘accessibility’ of the participants to the researcher (p. 201). The sample comprised two female third-year Bachelor of Education students with visual disabilities who acquired vision loss in early adulthood.

One participant was 21 and the other was 23. For one of the participants, the dust from the chalkboard exacerbated her visual challenges, while with the other, her challenges commenced with mild hearing loss followed by visual challenges. This participant used spectacles as a blending-in strategy (Goffman 1963) to conceal redness and swelling of the eyes. Only 2 of the 11 students in the learning community had visual disabilities. As an equity measure, both participants required print and electronic material in font size 14.

We selected the sample primarily because the participants were part of a teaching practice learning community, where SWDs receive support, guidance and skills for retention and success outcomes in their teaching practice school placements. Data were generated through semi-structured interviews to allow for rich understanding of the chalkboard experiences of students with visual disabilities. Prompts and probes were used to elicit relevant information and to extend conversations relevant to their experiences. This allowed the participants to narrate from their subjective positions rather than the researcher imposing meaning on their narration (Chase 2005). The interviews lasted approximately 60 min each which was sufficient to generate the required data. To ensure the transferability of the findings, detailed rich and thick descriptions of the participants’ voices are presented in the form of direct quotes (Denzin & Lincoln 2005).

Both the participants consented to their interviews being audio recorded. Data were transcribed verbatim from the original transcripts and audio recordings. Themes were derived using thematic analysis, which was prepared according to the procedures used by Braun and Clarke (2006). This involves familiarising oneself with the data; generating codes; searching for themes through the codes; reviewing and refining the themes; defining and naming them; and, finally, production of the research report. The benefit of using this method of analysis is that interpretation of the themes is supported by the data (Guest 2012). Although this method allowed categories to emerge from the data (Saldana 2009), it also runs the risk of missing nuanced data (Guest 2012).

### Ethical considerations

Prior to commencing this study, ethical consent was obtained from the relevant university (Ethical clearance no.: HSS/0108/014). In addition, gatekeeper’s permission was sought from the institution to undertake this study with students with visual disabilities. In noting the participant’s visual disabilities, consideration was accorded to print access in relation to reading the informed consent form and written feedback for member checking. The enquiry followed a rigorous process to uphold the trustworthiness and confidence of this investigation, including member checking, peer debriefing and thick descriptions of themes with direct quotations from the interviews (Denzin & Lincoln 2005).

Given the representation of third-year Bachelor of Education students with visual disabilities in the research site, as well as their representation in the general demographic of the student population, it must be emphasised that as authors, we make no claim to generalisability of the emerging findings, but we hope that useful indicators will be generated to influence the policy process for trainee teachers with visual disabilities. The purpose of this article is to highlight how structural constraints may be addressed pragmatically to enable student teachers with visual disabilities to experience equity of access, participation, inclusion and retention in their teaching practice school placements.

## Results and discussion

Based on the analysis of the data generated, the following experiences emerged. Barriers experienced in negotiating the chalkboard are considered, followed by the factors contributing to access. We use Tinto’s theoretical framework as an analytical lens to demonstrate that in the absence of adequate institutional support, both by the University and host institution, students resorted to shared knowledge, shared knowing, collaborative and cooperative learning as offered by learning communities and a teaching practice pre-placement booklet, as a hedge against the cultivation of ablest identities. In this study, the constructs of shared knowledge, shared knowing, collaborative and cooperative learning (Tinto 2003, 2004) stimulated claims for both barriers and factors contributing to access.

### Barriers to chalkboard access

Despite the support provided in teaching practice learning communities, students with visual disabilities experienced barriers to chalkboard accessibility and participation. These related to inadequate or no reasonable accommodations in under-resourced schools and imposing ablist^2^ identities on trainee teachers with disabilities.

#### Inadequate or no reasonable accommodations in under-resourced schools

In the absence of electricity, chalkboard access and participation were hindered because of visual disability intersecting with darkness in the classroom. Furthermore, overhead projectors or data projectors could not be used as a substitute for the chalkboard. A further complication related to the lack of resource materials such as chart paper to design posters as a substitute for the chalkboard. This is highlighted in the voices below.

‘In under resourced schools I only use the chalkboard. There are no lights there. What do you do? It is not easy to teach [*in under resourced schools*]. There are no lights. You cannot use the overhead projector or the data projector…’ (Thandi, 21 years old, visual challenges)

‘In under resourced schools …here you have to use the chalkboard and textbook. More often there is no lights so the class is dark this makes it difficult to write on the board… Also under resourced schools do not have chart paper to do posters, this becomes a further challenge.’ (Nellie, 23 years old, mild hearing loss and visual challenges)

The participants reported that these challenges limited their capability to perform effectively as trainee teachers. This is echoed by Glazzard and Dale (2015), who argue for specialised resources to improve retention and success in teaching practice school placements for students with dyslexia.

Given that South African higher education policy frameworks commit to broadening access and participation (Republic of South Africa 1997b, 2001, 2018), why have SWDs not been considered for the provisioning of equitable arrangements? The United Nations Convention on the Rights of Persons with Disabilities (CRPD 2006) draws attention to Article 2 on reasonable accommodation as a means of modifying or adjusting the environment to allow persons with disabilities to exercise their agency, human rights and individual freedoms on an equal basis with others.

On the one hand, as a human rights and social justice concern, policy initiatives call for inclusion, equal participation and the right to education for all. On the other, the current reality is that the absence or lack of reasonable accommodations in under-resourced schools remains a barrier to broadening access and participation in higher education for SWDs. This form of higher education disablist gaze marginalises students with visual disabilities in their teaching practice school placements and prevents the transformation and social change articulated in South African higher education policy frameworks (Republic of South Africa 1997a, 1997b, 2001, 2018).

#### Creating an ablest identity for chalkboard access

Inappropriate provisioning in under-resourced schools imposes an ablist identity on SWDs. Under the culture of ablism, discrimination against persons with disabilities is institutionalised, as they are assigned or denied skills and attributes. In order for the participants to negotiate the chalkboard, they downplayed their disability, to allow a sense of normalcy to prevail. They reported that they used large print prepared notes on A4 paper or spectacles to conceal their visual disability. This is highlighted in the following:

‘I do large print work sheets in preparation for class. Learners do not know my preparation is in large print. I do all this on A4 paper to create normality.’ (Nellie, 23 years old, mild hearing loss and visual challenges)

‘Even if learners laugh about your chalkboard writing, still be confident, be firm and serious. Don’t allow people to feel sorry for you. Do not look for sympathy, try and fit in. Use spectacles … so they think I am normal.’ (Thandi, 21 years old, visual challenges)

Assuming the normalist gaze within the context of disability discourses creates (a form of) disablism, where, through cultural, social and institutional norms and values, dominant groups impose a false identity to which SDWs must subscribe for acceptance and inclusion. Goffman (1963) claims that the structuring of persons with disabilities by ablist social structures creates the belief that they do not fit and are termed deviant. Hence, persons with disabilities have to blend in, to avoid stigma production (Goffman 1963). The participants in this study assumed false identities to ensure acceptance, inclusion and belonging in the classroom. Subrayen’s (2018) study also found that students with visual disabilities used blending-in strategies in their teaching practice school placements to reduce or avoid stigma production. These include, for example the use of spectacles to create an ablist identity. This finding resonates with the current study, as spectacles were used as a blending-in strategy to create a sense of normality. However, despite these barriers, through teaching practice learning community support, positive outcomes were achieved, as detailed below.

## Factors contributing to access and participation

### Reworking traditional methods of chalkboard access and participation through learning community support

The following voices summarise how the participants negotiated their visual disabilities by reworking the chalkboard to enhance retention and success. This finding points to the opportunities and potential offered by learning communities (Tinto 2003, 2004) to enhance retention and success in teaching practice.

As a strategy to broaden access and inclusion, the participants reworked traditional ways of accessing the chalkboard. For instance, one participant made use of the ‘Half-and-Half’ method to engage with the chalkboard. One half was used to stick large print posters from which the lesson was taught, while the other was used to write learner responses. This participant reported the use of different coloured chalk to negotiate chalkboard access. In this way, diverse aspects of the lesson were taught to the learners and the participant could also map out the entire lesson from beginning to end.

The other participant reported that for the purpose of interactive and collaborative classroom activity, the learners were asked to read her chalkboard writing.

‘I use the half and half method. I stick my prepared posters on the chalkboard and on the remaining part of the chalkboard I write the learners’ responses.’ (Thandi, 21 years old, visual challenges)

‘Using different coloured chalk helps me… Yellow [*chalk*] helps me because it is bright… I use yellow chalk for subheadings…I use white chalk for the content. Using different colour chalk for the content helps because it helps to know the beginning and the end of the unit. Yellow and white helps with my organisational ability on the chalkboard.’ (Thandi, 21 years old, visual challenges)

‘I write on the chalkboard and ask the learners to read. I tell the learners that I am here as a teacher so you can read for me so that you can understand what I wrote on the chalkboard.’ (Nellie, 23 years old, mild hearing loss and visual challenges)

Tinto (1975) claims that the nature of academic integration determines students’ retention and success or drop out. Although teaching practice learning communities are among the factors that contribute to retention and success of SWDs, the results of this study also suggest that such communities serve as a transition support structure. This allowed for the transportation of chalkboard access skills and strategies that included the participants’ visual disability skills. As social actors in teaching practice learning communities, students with visual disabilities bring with them specialised skills and knowledge about disability management and interventions required for retention and success in practice learning sites, which then become institutionalised in these sites.

In learning community student group programmes, students typically share experiences relating to school visits; adaptation to the school environment; professional relationships and interventions to support teaching practice learning experiences. These engagements are used to orientate and socialise students before their teaching practice placements. This allows for inclusion, retention and success. The somewhat negative outlook for teacher trainees with visual disabilities presented earlier, particularly with regard to the use of visual teaching aids, is mitigated by shared knowing, shared knowledge and the collaborative and cooperative learning afforded by learning communities. Finally, we also found the teaching practice pre-placement booklet (itself a product of the learning community) to be a useful resource to support transition, retention and success in teaching practice school placements, as elucidated below.

### Teaching practice booklet for an effective teaching practice school placement

Amidst the generally pessimistic outlook for SWDs, the findings of this study highlight an effective strategy, particularly in the absence of substantive support structures for SWDs. The participants viewed the booklet on ‘Management Strategies for an Effective Teaching Practice Placement for Students with Visual Disabilities’ as a retention tool that enabled them to use disability specific trajectories from the booklet in teaching practice school contexts. This enhanced their chalkboard access and participation, hence achieving successful chalkboard experiences, as revealed in the following extracts from the interviews with the research participants

‘… the booklet developed by the disability unit also helped and made me confident. This booklet made me ready for teaching practice in a way that if a challenge happens then I will know what to do. You manage your challenges because of support.’ (Nellie, 23 years old, mild hearing loss and visual challenges)

‘I also used the booklet…it has all the strategies I need to use. I put all in place resulting in an effective teaching practice placement. This creates a positive learning experience. This makes me confident to manage my challenges.’ (Thandi, 21 years old, visual challenges)

These excerpts show that the booklet improved confidence, planning and preparedness and assertiveness, which promoted positive learning experiences. Participants’ strategies contributed to a pedagogy of nuanced possibilities in their teaching practice school placements. Other studies also highlight good practice measures to enhance the practice learning placements of SWDs, including the six-phase tripartite model developed by Griffiths et al. (2010); structured pre-practice placement meetings between all practice partners and the student (Ashcroft et al. 2008); and technological aids to support trainee teachers with dyslexia (Glazzard & Dale 2015). More recently, good practice measures were documented in Subrayen’s (2018) study that supports Tinto’s (2003, 2004) Learning Community Model as an institutional framework to enhance equity, access and participation in teaching practice school placements of SWDs. This was found to be critical for the achievement of equality.

## Concluding observations

The study from which this article derives examined the chalkboard experiences of Bachelor of Education students with visual disabilities in their teaching practice school placements. Several themes emerged which signalled overt and covert barriers to access and participation. Aside from these barriers, this study also signals that reworking traditional methods of chalkboard access together with a pre-placement teaching practice booklet for SWDs enhanced the retention and success outcomes of trainee teachers with visual disabilities. The findings also demonstrate the value of transition and retention support trajectories such as teaching practice school placement learning communities. One of the more significant findings relates to flexible chalkboard teaching and learning designs such as large print posters, using the chalkboard and posters simultaneously, and using different coloured chalk. In addition to teaching practice school placement learning communities for students with visual disabilities, the teaching practice booklet served as a resource or toolkit to minimise barriers to chalkboard access and participation.

Taken together, the findings suggest that despite redesigning access and participation in relation to the chalkboard, complexities and challenges persist. This raises the need for policy review and for the higher education sector to consider systemic structures for retention and success. As Tinto (2006–2007:5) argues, we need to look at ‘what works’ to enhance retention and success for diversified student cohorts. There must be a call for deeper structural connections with institutional climates and, through systemic transformation of higher education policy frameworks, there is a need to develop structured teaching practice learning communities for SWDs.

Although the data and analysis provide snapshots of the participants’ accounts of their experiences, these were not corroborated by observations or other methods and sources. What the participants say may or may not be very different from the way they actually ‘negotiate’ the chalkboard in the classroom, especially as trainee teachers. Additional methods and sources would have enhanced the trustworthiness of the research. This is acknowledged as a limitation, and it is hoped that subsequent studies will embrace a wider range of research methods and instruments to corroborate the findings and analyses offered here.

Finally, higher education institutions have an obligation to ensure that teaching practice school placement assessors undergo formal and extended learning in relation to disability in higher education and non-traditional teaching and learning strategies and interventions used by SWDs in their teaching practice school placements. Assessors are also advised to be cognisant of contextual differences in resourced and under-resourced schools and the diverse ways in which SWDs navigate these differences in their teaching practice school placements.

## Footnotes

1 A substantive discussion of the constructs of equity, access and participation can be found at: http://unesdoc.unesco.org/images/0024/002482/248254e.pdf.

2 Ablism refers to discrimination against people with disabilities who are considered inferior to the non-disabled.
